# High serological barriers may contribute to restricted Influenza-A-virus transmission between pigs and humans

**DOI:** 10.1016/j.onehlt.2025.101214

**Published:** 2025-10-14

**Authors:** Christin Hennig, Annika Graaf-Rau, Kathrin Schmies, Roland Elling, Philipp Henneke, Ralf Dürrwald, Elisabeth grosse Beilage, Martin Schwemmle, Martin Beer, Timm Harder

**Affiliations:** a Institute for Diagnostic Virology, Friedrich-Loeffler Institute, Greifswald-Insel Riems, Germany; b Helmholtz Institute for OneHealth, Greifswald, Germany; c Field Station for Epidemiology, University of Veterinary Medicine Hannover, Bakum, Germany; d Center for Pediatrics and Adolescent Medicine, and Institute for Immunodeficiency, Center for Chronic Immunodeficiency, Medical Center, Faculty of Medicine, University of Freiburg, Freiburg, Germany; e Institute for Infection Prevention and Control & Institute for Immunodeficiency, Medical Center of Freiburg, Freiburg, Germany; f National Influenza Center, Unit 17 Influenza and other respiratory viruses, Department of Infectious Diseases, Robert Koch-Institute, Berlin, Germany; g Institute of Virology, Medical Center - University of Freiburg, Freiburg, Germany; h Faculty of Medicine, University of Freiburg, Freiburg, Germany

**Keywords:** Influenza A virus, Swine influenza virus, Zoonosis, Spillover infection, One-Health, Anthropozoonosis, Zooanthroponosis, Human-swine interface

## Abstract

Influenza A viruses (IAV) circulate in both humans and pigs, with bidirectional transmission potentially driving viral evolution. Despite frequent contact and genetic compatibility, observed cross-species transmission remains rare, suggesting the presence of unexplored or little-known barriers. The study investigated transmission dynamics and mechanisms restricting IAV spread at the human-swine interface in Germany. We analyzed 3070 porcine and 333 human nasal swabs from 135 swine farms via RT-qPCR and full-genome sequencing. Concurrently, we conducted serological surveys: 1) Children's sera (urban, no pig contact) for antibodies against circulating swine IAV, and 2) Swine sera for antibodies against human-adapted IAV. Molecular surveillance identified only one zooanthroponosis event and sporadic anthropozoonosis (primarily in children) despite swine IAV strains carrying zoonotic-propensity genetic markers (MxA resistance). Serologically, urban children without pig exposure exhibited marked neutralizing activity against swine IAV, whereas swine sera contained neutralizing antibodies against human IAV strains. Pre-existing cross-reactive immunity—evidenced by unexpected antibody prevalence in both species—creates a more complex interspecies barrier than genetic factors alone. This serological “shield” may critically limit IAV transmission between humans and pigs, reshaping our understanding of zoonotic risk.

## Highlights

• IAV with zoonotic propensity circulate in swine populations in Germany.
• Multifaceted investigations revealed a complex human-swine interspecies barrier.
• Serological barriers may restrict IAV transmission between pigs and humans.
• Continuing surveillance of IAV in swine remains mandatory.

## 1 Introduction

Influenza A viruses (IAV) cause seasonal outbreaks and sporadic pandemics in humans, with significant global health impacts [1,2]. While birds, and particularly waterbirds, are primary reservoirs, pigs serve as critical “mixing vessels” for IAV evolution due to their susceptibility to both human and avian strains [3]. Cross-species transmission between animals, including those of different taxonomic classes (e.g., mammals and avians) and humans remains a persistent threat to public health and animal welfare [4]. The pivotal role of pigs as ‘mixing vessels’ for IAVs was unequivocally demonstrated in 2009 when a quadruple-reassortant swine-origin virus (A/H1N1pdm09) caused a pandemic in humans. The 2009 IAV strain emerged from the recombination of Classical North American swine H1N1 (HA, NP, NS segments), Eurasian avian-like swine H1N1 (NA, M segments), North American triple-reassortant swine H3N2 (PB2, PA segments), and Human seasonal H3N2 (PB1 segment) [[5], [6], [7]]. An initial zoonotic spillover of swine influenza A viruses (swIAV) into humans (anthropozoonosis) may seed human-to-human transmission chains. This transmission depends on direct or indirect contact at the human-swine interface [8]. Seroprevalence data confirm that individuals with occupational swine exposure show significantly higher swIAV antibody levels than the general population, demonstrating their elevated risk of zoonotic swIAV exposure [9,10].

Human-to-pig transmission (zooanthroponosis, also termed ‘reverse zoonosis’) is the primary source of IAV diversity in global pig populations. [11] Historical human pandemic viruses (except H2N2 in 1957/8) and seasonal strains have established stable lineages in pigs. In contrast, only a few spillover events from avian sources have resulted in stable influenza lineages in swine populations. The most prominent instance emerged in Europe during the late 1970s, leading to the establishment of the H1 swIAV clade 1C, which is also referred to as the European avian-derived lineage [[10], [11], [12], [13], [14]]. Another instance of avian-to-swine spillover within North America has resulted in the emergence of swine influenza A virus (swIAV) reassortants, which harbor the so-called TRIG cassette comprising internal genome segments. These reassortants have played a significant role in the emergence of the A/H1N1pdm2009 pandemic virus [15]. In Germany, as well as throughout the majority of the European mainland, the subtypes H1N1, H1N2, and H3N2 have established enduring lineages within porcine populations. Three distinct lineages of the H1 subtype have been identified: swIAV of avian (av) origin (H1C) accounts for the majority of swine infections in Germany, constituting approximately 60–70 %, whereas swIAV of human (hu) origin (H1A = A/H1N1pdm2009 and H1B) contributes 10–20 % (H1A) and 5–10 % (H1B), respectively. The H3 subtype has been detected at a low frequency [5,6]. The increasing diversity of novel genotypes within these lineages due to interlineage reassortments has facilitated the formation of influenza A virus (IAV) reservoirs with indeterminate zoonotic potential in pigs located in Germany and other regions [5,[16], [17], [18]]. Once established as a stable lineage in porcine hosts, both human- and avian-derived IAV, along with their reassortants, have been demonstrated to induce respiratory diseases, which adversely affect animal welfare and lead to significant production losses [6]. In swine operations housing more than 3000 animals, the continual influx of susceptible individuals from newly born piglets ensures the persistent circulation of IAV within these facilities. The enzootic status of swIAV has been shown to accelerate antigenic diversification, which may ultimately culminate in the emergence of antigenic variants capable of evading vaccine-mediated control in pigs [18,19].

During the period spanning 2007 to 2020, a cumulative total of six documented cases of anthropozoonotic swine influenza A virus (swIAV) infections in humans within Germany were identified. These involved three pediatric patients, one individual with compromised immunity, and two adults who were previously considered healthy [20,21]. In four of these cases, either direct or indirect exposure to swine was evidenced, whereas in two instances, no such contact was reported. The clinical manifestations presented by all affected individuals were congruent with mild to moderate influenza-like illness (ILI). No onward human-to-human transmissions were observed [20,22]. Consistent with the majority of sporadic swIAV infections documented in human subjects throughout Europe, the majority of cases displayed a benign clinical trajectory that was indistinguishable from upper respiratory tract infections attributed to various other causative agents. Consequently, the potential that such spillover incidents occur with greater frequency, yet remain undiagnosed, cannot be dismissed [23,24]. Research has established that children demonstrate an enhanced vulnerability to both seasonal and pandemic influenza A virus (IAV) compared to adults, and they play a significant role in the transmission dynamics of IAV within the community [25,26]. Therefore, children may also serve a critical function in the initial acquisition and subsequent propagation of zoonotic swIAV [26].

Here we undertook virological surveillance of porcine populations and personnel across 135 swine enterprises in Germany from September 2021 to October 2023. The investigation focused on potentially adaptive mutations that facilitate infection in the human host by circumventing the human MxA barrier, as observed in swIAV sequences derived from the surveillance initiative, as well as in two recent instances of zooanthroponotic transmission in humans identified independently within Germany [21]. Furthermore, a cohort of pediatric sera from children lacking any exposure to pigs was analyzed for the presence of cross-reactive antibodies against swIAV strains circulating among swine in Germany. Simultaneously, sera from swine were assessed for antibodies that cross-react with seasonal human IAV variants.

## 2 Material and methods

### 2.1 Collection of human and swine nasal swab samples

The study was open to pig farmers and specialised swine veterinary practices [27]. Inclusion criteria for participation were based on a history of an swIAV outbreak in the swine holding or a currently suspected swIAV infection, and the (written) consent to provide human and swine nasal swab samples.

Samples from swine holdings were taken from individual pigs on a strictly diagnostic base by farmworkers or veterinarians, instructed to take samples from pigs with clinical signs of a respiratory disease (e.g. dyspnea, coughing, nasal discharge, fever). At the same time, at least one person with close contact to the sampled pigs (e.g. farmworker, veterinarian) or family members of the staff was asked to contribute a nasal swab sample from themselves. Further specifications were determined in a questionnaire (age, time of year, presence of ILI symptoms, vaccination status, etc.) which are accessible in the data pool in the Zenodo repository (see Table S2) [28]. A detailed description on self-sampling a nasal swab as well as appropriate sampling material were provided. Human and swine samples were received from pig holdings in Germany between September 2021 and October 2023.

Information about each swine holding were retrieved in a questionnaire [28], in which age of sampled pigs, clinical signs in the herd, swIAV detection history, vaccination status and information about the farm structure (specialization, herd size) were recorded (see Table S1) [28]. The study design was approved by the ethics commission of the University of Greifswald, Germany (approval number BB095/20). All human participants signed informed consent forms and had the option to withdraw their sample from this study at any time.

During this study, researchers documented two separate zoonotic swIAV transmissions in Germany. In the first case (2021), a 17-year-old swine farm trainee in Mecklenburg-Western Pomerania (MWP) contracted subtype H1CN1 (MWP/21; EPI_ISL_2434153). We isolated a genetically matching swine strain (sw-MWP/21; EPI_ISL_17646374) from diseased pigs at the same holding. In the second case (2022), an adult in North Rhine-Westphalia (NRW) with an influenza-like illness tested positive for swIAV H1N1 (NRW/22; EPI_ISL_12589314), but there was no verified swine contact [21].

### 2.2 Serum samples

Serum aliquots from 75 children and adolescents (2–18 years of age) from Freiburg, Germany, had been collected in May 2020 during a SARS-CoV-2 household transmission study [28,29]. These sera could be used due to a broader ethics approval (University of Freiburg: 256/20_201553) but only the participants' age was disclosed. Swine sera (*n* = 40) of pigs in Germany were collected as part of the European ICRAD “PIGIE” project (number 2821ERA24) from holdings (i) where sows had been vaccinated against swIAV (*n* = 3) or (ii) where acute swIAV infections had been detected by RT-qPCR (n = 4).

### 2.3 Origin of reference viruses

Swine influenza A viruses (swIAV) used as references here were obtained from collections at the Friedrich Loeffler-Institute (FLI), Isle of Riems, Germany. Human influenza A viruses (huIAV) representing seasonal vaccine strains, were provided by the National Influenza Centre at the Robert-Koch-Institute (RKI), Berlin, Germany.

### 2.4 Viral RNA extraction and Real time RT-PCR (RT-qPCR)

RNA extraction was performed either manually by using the QIAamp Viral RNA Mini Kit (Qiagen, Hilden, Germany) or automatedly with the King Fisher Flex Purification System (Thermo Fisher Scientific) and the NucleoMag® Vet Kit (Macherey-Nagel GmbH & Co. KG, Dueren, Germany).

Swine samples were tested in a triplex-pathogen RT-qPCR assay probing simultaneously for swIAV (M gene target), porcine respirovirus-1 (PRV-1) and swine ortho-pneumovirus (SOV) [5]. Sample positive for swIAV (cq-values ≤39.9) were subtyped in a multiplex RT-qPCR assay as previously described distinguishing five HA lineages (H1av [1C], H1hu [1B], H1pdm [1 A], H3–84, H3–04) and three NA subtypes or lineages (N1av, N1pdm, N2) [5]. Human samples were analyzed exclusively for IAV by a generic M gene-specific RT-qPCR [31]. All RT-qPCR reactions were prepared with the AgPath-ID™ One-Step RT-qPCR kit (Thermo Fisher Scientific, United States) and run on a Biorad CFX96 Real-Time Cycler (Biorad, Germany).

### 2.5 Virus isolation in cell culture

Virus isolation was attempted for RT-qPCR-positive samples with a cq-value of ≤30. Madin-Darby-Canine kidney cells (MDCK-II) or swine testicle (ST) cells (BioBank at FLI, no. 0606) were employed as described previously [5].

### 2.6 Sequencing of swIAV genomes

Sanger sequencing was used to generate sequences of the HA IAV-gene from samples with cq- values ranging from 25 to 32 [5]. Field samples with cq-values ≤25 were selected for whole genome sequencing by Oxford Nanopore technology as described previously [32]. Sequences obtained were deposited in the EpiFlu database of GISAID. Accession numbers are presented in Table S2.

### 2.7 Phylogenetic analyses

HA segment-specific multiple alignments were generated using MAFFT (v7.450) [33] and manually curated and trimmed by AliView [34]. Phylogenetic estimations were carried out by maximum likelihood (ML) algorithms implemented in IQTree [35] utilizing IQTree's ModelFinder to select the most appropriate model according to the Bayesian informative criterion [30]. Robustness of consensus trees was probed using UFBoot [36] and trees were visualized with FigTree (V1.4.4) (http://tree.bio.ed.ac.uk/software/figtree/) and further manually edited with Inkscape (https://inkscape.org/). In addition, HA lineages and clades were determined using the BV-BRC tool [37] accessible via the BV-BRC website (www.bv-brc.org/app/SubspeciesClassification).

### 2.8 Genotyping

Genotyping was conducted following the approach of Graaf-Rau et al. (2023) [5] by aligning full length segmental swIAV sequences to reference sequences.

### 2.9 Molecular *in silico* analyses

The Flusurver online tool (http://flusurver.bii.a-star.edu.sg) was used to detect and analyze mutations in the IAV genome. Neutralization-relevant epitopes in deduced HA1 protein sequences of swIAV and human IAV were compared according to Sun et al. [10]. Alignments were generated with MAFFT using the Geneious software version 2021.0.1 and further processed with WebLogo [38] and Biorender.com (https://www.biorender.com/). Mutations in the NP protein that interfere with Mx and BTN3A3 factors were identified according to Henritzi et al. [6] and Pinto et al. [39].

### 2.10 Enzyme linked immunosorbent assay (ELISA)

Human and porcine serum samples were heat-inactivated (56 °C for 30 min) before first use. For the human sera, the ab108745 - Anti-Influenza virus A IgG Human ELISA Kit (Abcam®, Cambridge, United Kingdom) was used to detect IgG antibodies against IAV. The swine sera were tested for IAV NP-specific antibodies by the ELISA kit ID Screen Influenza A Antibody Competition Multi-species (IDvet®, Grabels, France).

### 2.11 Virus neutralization assay (VNT)

Heat-inactivated human and swine serum samples were treated with neuraminidase as previously described [6]. An MDCK-II cell-based method was used essentially as described by Henritzi et al. [6]. Briefly, serum samples were serially diluted twofold, starting at 1:20, in DMEM medium supplemented with 6-(1-tosylamido-2-phenyl)-ethyl-chloromethyl-ketone (TPCK)-treated trypsin (infection medium) at a final concentration of 1 μg/mL. Viruses used in this study, were diluted in infection medium to a concentration of 2000 TCID_50_/ml (10^3,3^ TCID_50_/mL). At a volume of 50 μL each, diluted serum and virus were mixed and incubated for 1 h at 37 °C before being transferred to the MDCK-II cell plates. The plates were incubated at 37 °C for 72 h, after which cytopathic effects (CPE) were read by light microscopy. Standard negative and positive sera raised in ferrets were used as controls.

## 3 Results

### 3.1 Moderate incidence of swIAV infections in pigs in swine herds in Germany

**Fig. 1:**
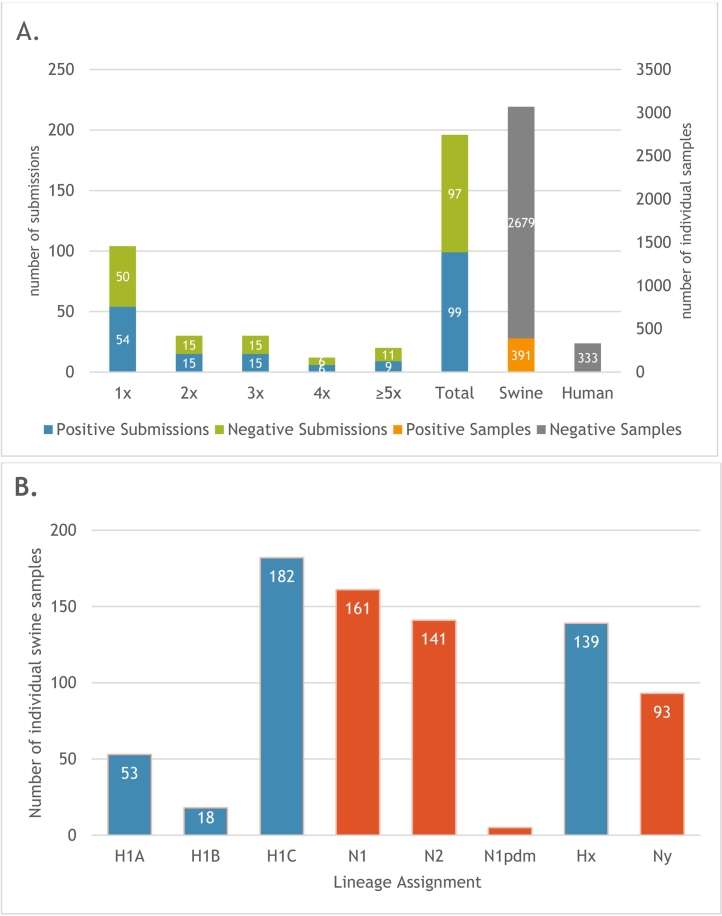
Detection of swine influenza A virus infections in pig holdings and human staff in Germany. A. Frequency of repeated sampling in pig holdings, and total individual porcine and human samples (x-axis). B. Frequency distribution of hemagglutinin (H) subtype H1 and neuraminidase (N) lineages in pigs; Hx/Ny – no subtype/lineage was assignable due to low virus concentrations in the samples.

A total of 196 submissions from 135 different swine holdings were received. A history of previous swIAV outbreaks was documented in 64.8 % of the farms surveyed. In a total of 3070 individual porcine samples, a generic M-gene-specific RT-qPCR detected 391 (12.4 %) swIAV positives (see Fig. 1A and Table S1) [40]. Weaned and suckling piglets (15.1 % and 14 %, respectively) exhibited higher IAV detection rates compared to all other age groups. A higher frequency of swIAV detection was observed in farms housing young pigs. Among the 160 submissions from farms reporting respiratory signs or reproductive distress, 93 (58.1 %) tested positive for swIAV. Conversely, a mere 6.6 % of farms with a documented history of swIAV, devoid of present clinical indications, tested swIAV positive. Of note, 79 of the 99 swIAV-positive submissions (79.8 %) occurred despite the use of commercial vaccines for sow vaccination.

### 3.2 A wide variety of swIAV H1 lineages and genotypes circulated in swine holdings in Germany

Representatives of three swIAV H1 lineages (1 A, 1B, and 1C) were detected (Fig. 1B). At the farm level, the most prevalent subtype identified was H1CN1 (35.5 %), followed by H1CN2 (10.5 %) and H1BN2 (6.6 %). The prevalence of H1AN1 and H1AN2 was identified in 5.3 % and 2.6 % of the cases, respectively. The H3N2 subtype was detected in a single holding only. An analysis of 37 whole genome sequences detected 13 different genotypes (Table 1). Notably, the genotype “AV” had not been previously documented [6]. The “pure” genotype (“A”) of the avian-derived H1CN1 lineage constituted the majority of the genomes (*n* = 11), while “pure” human pandemic A/H1N1 2009 (“P”) was represented by a single genome only. The human-derived sequence of A/New York/PV00909/2018 was identified as the closest related sequence to the porcine genotype “P” sequences (97 % nucleotide identity). This suggests a recent zooanthroponotic spillover incidence (Fig. S1).

**Table 1:**
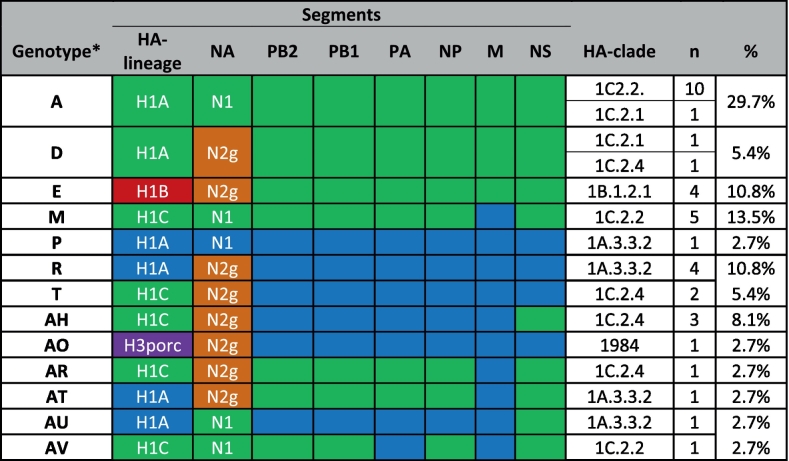
Subtype and genotype variation of swIAV according to full genome sequence analyses. Genome segments phylogenetically associated with the avian-derived H1 (1C) lineage are colored green, those of the human pandemic A/H1N1 2009 lineage (1A) are shown in blue. HA subtype 1 clades are labelled according to Andersson et al. [13]. H3porc (purple) indicates similarity with A/Port Chalmers/1/73 (H3N2)-like viruses (clade “1984”). N2g (orange) indicates close relationship with A/sw/Gent/1/1984-like swIAV.

*Genotype identification was according to Graaf-Rau et al. [5].

Two recent anthropozoonotic human swIAV infections, MWP/21 and NRW/22, which were reported independently from Germany, were assigned to genotype A (Table 1). Their HA gene clustered with clade 1C.2.1 (MWP/21) and 1C.2.2 (NRW/22), respectively (Fig. S1).

### 3.3 No active IAV infections in human study subjects despite low influenza vaccination status

The study comprised 226 human participants (working on the swine holdings under surveillance) who submitted a nasal swab sample once (*n* = 169/226), twice (*n* = 37/226) or ≥ 3 times (*n* = 20/226), resulting in a total of 333 human samples. The majority of the participants were farm workers (61.1 %; *n* = 138). Veterinarians (13.3 %; *n* = 30) and family members of farm workers (12.8 %; *n* = 29) or veterinarians (0.9 %; n = 2) submitted further samples; 11.9 % of the participants did not provide information. We collected 14 samples from persons younger than 25 years (6.2 %), while the majority of the participants was aged 25 and 60 years (60.2 %), 11.1 % were over 60 years of age; 22.6 % did not disclose their age. At the time of sampling, 93.4 % of the participants self-reported feeling healthy. Tables 2 and S1 present characteristics about the participants' seasonal IAV vaccination status: A minority of 47/226 (20.8 %) received regular annual seasonal IAV vaccination. IAV was not detected in any of the human samples although 143/333 (42.9 %) were provided by participants from farms with swIAV-positive pigs at the time of sampling. This finding suggests potential exposure to swIAV (Table 2, S1). Likewise, IAV was not detected in samples from 22 human participants (6.6 %) who reported ILI.

**Table 2:** Seasonal influenza vaccination status of human study subjects and their potential exposure to swIAV.

Vaccination status	Number of participants	%	Human samples from swIAV positive farms	%
None	114	50.4 %	78	54.5 %
Yes - regularly	47	20.8 %	30	21.0 %
Yes - irregularly	34	15.0 %	21	14.7 %
Not reported	31	13.7 %	14	9.8 %
Total	226	100	143	100

### 3.4 swIAV circulating in pigs or detected in two recent anthropozoonotic human swIAV infections reveal MxA and BTN3A3 escape mutations

MxA and BTN3A3 were reported to constitute major barriers shielding zooanthroponotic human IAV infections [6,41]. All previous pandemic IAV developed mutations that aided in MxA escape [52]. Since previous studies have indicated the presence of such escape mutation in swIAV from Germany, we concentrated on these mutations in the analysis of further swIAV sequences generated here. Various combinations of amino acid (AA) substitutions associated with human MxA escape were identified (Table 3). Sequences of two recent anthropozoonotic human swIAV infections from Germany, MWP/21 and NRW/22, exhibited four substitutions relevant for MxA- and one for BTN3A3-escape. This pattern was replicated in 10 additional porcine sequences, including the porcine precursor sequence of MWP/21 that was identified in the frame of this study (see Table 3).

**Table 3:**
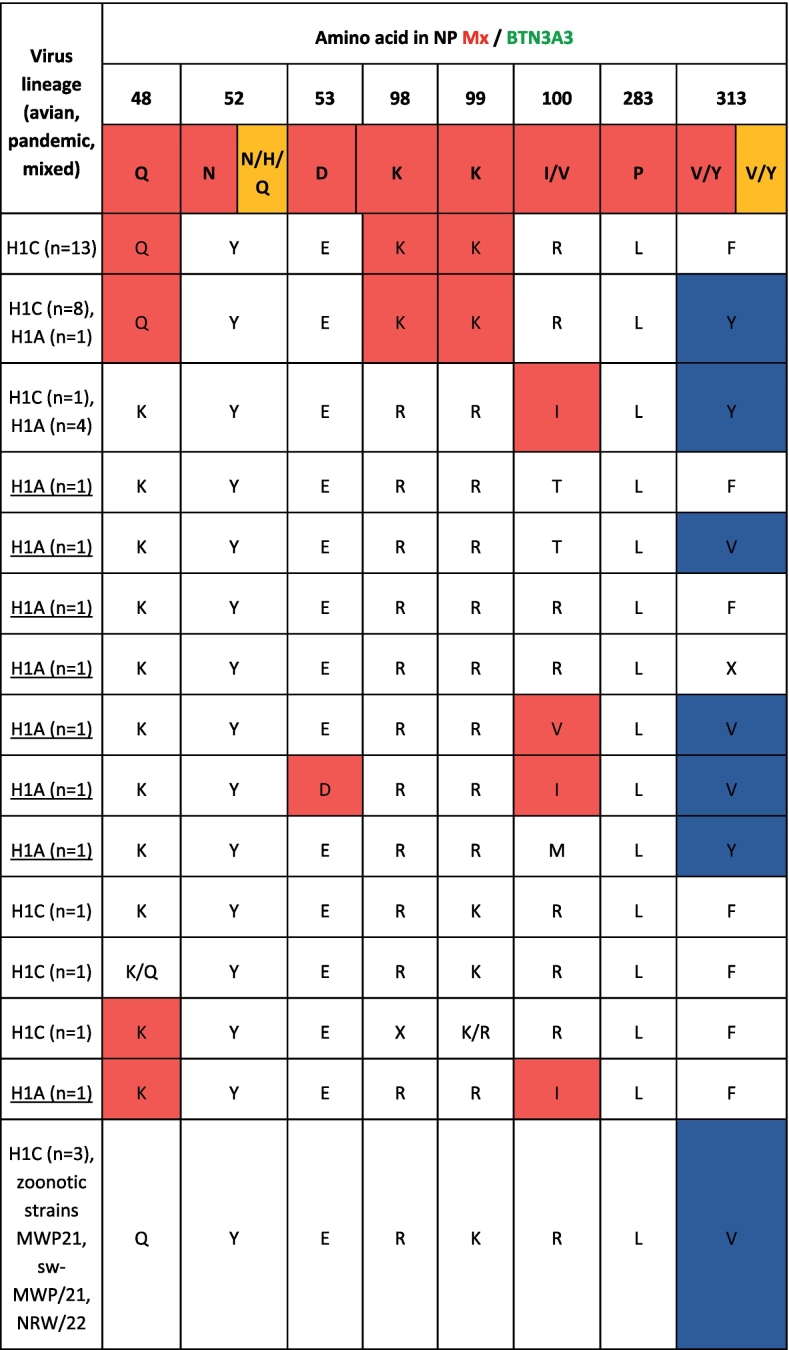
Summary of AA substitution patterns in the nucleoprotein (NP) related to human MxA and BTN3A3 restriction identified in this study in porcine and two human-origin swIAV NP sequences.

Red cells signal MxA escape mutations, orange color depicts BTN3A3 escape.

### 3.5 Sera of urban children without pig exposure show variable neutralization titers against selected circulating swIAV strains

**Fig. 2:**
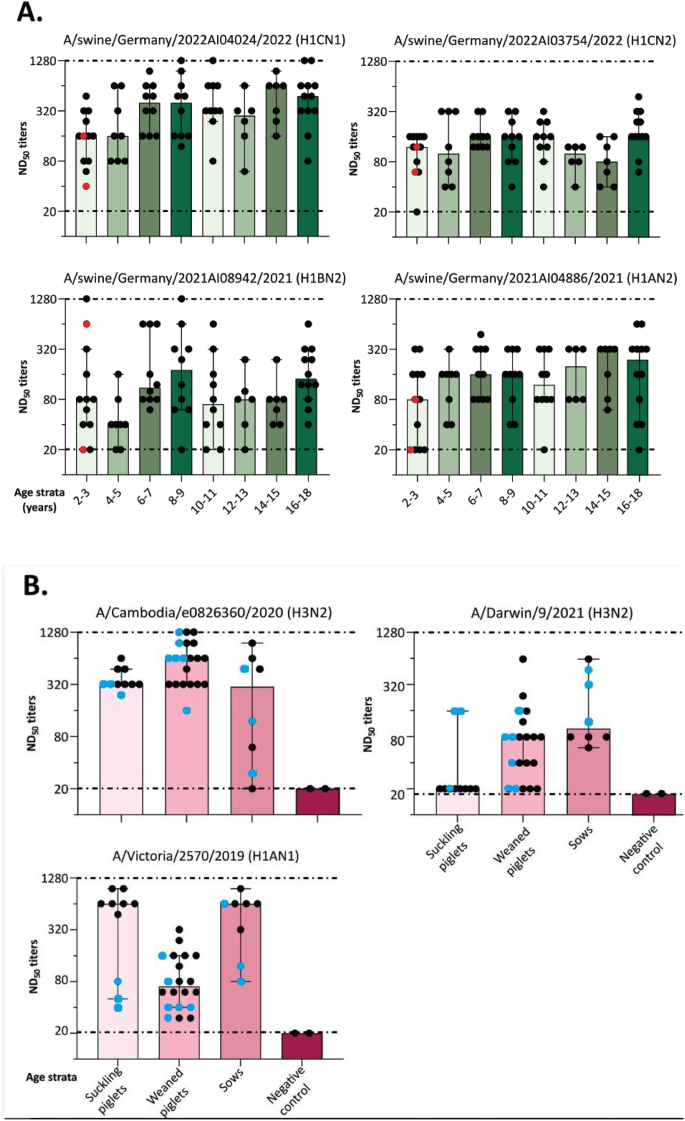
Neutralization of IAV strains by human (A) and swine (B) serum samples. Each point represents an ND_50_ titer of an individual serum, bars indicate geometric mean ND_50_ titers, black error bars represent 95 % CI. Sera with a titer of 1:20 or lower were considered negative (lower dashed line). Titres were not further specified if ≥1280 (upper dashed line). Children sera (A) are stratified by age (years); swine sera (B) are grouped by age classes. Red dots (A) indicate the two children who tested negative in ELISA, although showing neutralizing capacity in NT. Blue dots (B) indicate sera that originated from non-vaccinated animals. (For interpretation of the references to color in this figure legend, the reader is referred to the web version of this article.)

A cohort of 75 sera from children and adolescents aged 2–18 years was analyzed for its reactivity with swIAV [28,29]. An ELISA assay specific for IgG antibodies was utilized, revealing that with the exception of two samples from two-year-old children, the remaining samples exhibited antibodies against IAV. All sera were then tested by virus neutralization against swIAV strains representing different subtypes and lineages that were currently circulating in pigs in Germany including H1 AN2, clade 1 A.3.3.2, H1BN2, clade 1B.1.2.1, H1CN2, clade 1C.2.4, and H1CN1, clade 1C.2.2 (Fig. S1). The neutralizing capacity of the sera was evident in the majority of cases against all swIAV strains that were tested (Fig. 2A). The highest neutralization titers (NT) were observed against subtype H1CN1, with a median NT of 360 across all age groups. The subtypes H1CN2 and H1 A2 were efficiently neutralized with median NTs of 140 and 160, respectively. The lowest observed NTs were associated with the H1BN2 subtype, with a median NT of 90. The two children who were tested negative by ELISA still demonstrated neutralizing capacity against some of the tested swIAV (Fig. 2A, highlighted in red). The highest number of neutralization-negative sera was found in the group of 2–3-year-old children (*n* = 4/12) with NTs of ≤20 against subtypes H1 AN2 and H1BN2. However, human subjects with NTs ≤ 20 were also observed across various age groups and other reference viruses (Fig. 2A).

### 3.6 Swine sera show variable cross neutralization activity against current human IAV

A total of 35 pig sera positive for swIAV NP-specific antibodies by ELISA were examined for their neutralizing potency against recent human seasonal vaccine strains A/Darwin/9/2021 (H3N2), A/Cambodia/e0826360/2020 (H3N2), and A/Victoria/2579/2019 (H1N1). These vaccine strains have been in use in the human community during the IAV seasons 2021/2022 and 2022/2023, respectively [42]. Five sows of the panel were found to be seropositive following vaccination with Respiporc® FLU3 and FLUpan H1N1 swIAV vaccines (Ceva Santé Animale, France), while none of the other seropositive pigs had received vaccination. The neutralizing potency of swine sera exhibited significant variation between the two H3N2 strains that were examined (Fig. 2B; black dots indicate vaccinated animals or suckling piglets with MDA from vaccinated sows). While sera from suckling piglets exhibited low titers for A/Darwin/9/2021, the titers for A/Cambodia/e0826360/2020 were markedly higher. These trends were consistent across other age classes. High NTs were observed against the human H1 A seasonal vaccine strain A/Victoria/2570/2019 in sera from sows vaccinated against H1 AN1 (FLUpan) and H1CN1 (FLU3) and their associated, MDA-positive suckling piglets. Lower titers were observed in sera from unvaccinated sows and their suckling piglets. In comparison to the other groups, weaning piglets demonstrated lower overall titers.

## 4 Discussion

The actual frequency of swIAV transmission across the swine-human interface remains unknown. Our surveillance in 135 swine farms gave evidence for only one human-to-pig transmission, of a clade 1 A.3.3.2 virus. Two pig-to-human transmissions of swIAV of the H1C lineage were reported independently during the period of our surveillance study and outside of the farms initially included in our study [20,21]. A broad and genetically diverse spectrum of swIAV lineages was found to circulate in swine holdings. This provides opportunities for occupational exposure through aerosols, direct and indirect contacts. Yet, no pig-to-human transmission was detected in swine farm staff in the infected swine herds examined here. Nevertheless, the possibility that human swIAV infections were overlooked in this study cannot be fully dismissed: The quality of the self-swabbing technique used by the human participants and sample delivery were not controlled. Also, samples were taken at the volunteer's consent, which may have resulted in missing the optimal time point to capture swIAV excretion. However, technical issues post sampling, such as inadequate sensitivity of RT-qPCRs, are dismissed as explanations for the potentially overlooked presence of human swIAV infections. We did not attempt to detect IAV in aerosols at farms as a predictor of exposure. Despite the increasing significance of detecting IAV in aerosols, nasal swabs continue to be the most sensitive sample for identifying IAV infection [43].

The low transmission rate at the human-swine interface may also reflect reduced susceptibility in the study participants to swIAV, likely due to pre-existing immunity. Previous studies show that individuals with prior swine exposure develop higher antibody levels against swIAV than unexposed individuals [9,44]. Based on this data and considering that the majority (60,2 %) of human subjects of this study were farm workers aged between 25 and 60 years, pre-existing immunity to IAV can be assumed in this cohort. Furthermore, frequent occupational contact with swIAV might have broadened their IAV-specific immunity. In line with this assumption are previous observations of extended neutralizing capacity of human adult sera against recent swIAV circulating in Europe [6,9,45]. However, diagnostic proof was not possible in the present study since no serum samples were obtained from the subjects. .

Although incidences of swine-to-human IAV transmission in Europe as well as in North America are sporadic, the involvement of children, adolescents or immunocompromised patients is disproportionately high [24]. This suggests a higher likelihood of children being susceptible to anthropozoonotic swIAV infections. Seroprevalence for swIAV in children and adolescents has been rarely analyzed [44]. Sauerbrei et al. [46] assume that around one third of under six-year-olds in Germany has never had contact with IAV. Children and adolescents therefore could play a role as primary susceptible targets, that amplify and spread potentially zoonotic swIAV upon exposure. The urban cohort of children and adolescents aged 2–18 years (*n* = 75) examined here demonstrated high prevalence of antibodies neutralizing swIAV (Fig. 2A).

Sauerbrei et al. [46] indicated that IAV transmissions are more prevalent in children and that children exhibit a more robust serological reaction. This may have been a contributing factor to the observed reactivity pattern. The impact of prior influenza A virus (IAV) infections or vaccinations could not be evaluated due to an absence of data on this subject. The pediatric sera had been collected after the cessation of a human influenza season, at a time when both subtypes of seasonal IAV (A/H3N2) and (A/H1N1pdm09) were circulating in parallel. Serologically naïve individuals were restricted mainly to the age group of 2–3-year-olds and, individual sera lacking neutralizing capacity against at least one of the swIAV strains tested were present in almost all other age groups.

The pediatric sera also suffer from a potential selection bias as they are repurposed from a SARS-CoV-2 study; however, there are no cross reacting antibodies between SARS-Cov-2 and influenza A viruses. Adult sera have been examined against a similar set of swIAV from our lab in a previous study with similar results, i.e. a high prevalence of cross-reactive neutralizing antibodies [6].

Given the established role of human IAV incursions into swine populations in expanding the IAV reservoir in pigs, we examined 40 sera obtained from pigs of different age strata against human seasonal IAV vaccine strains. The tests revealed that sows vaccinated with the FLUpan vaccine, which contains a pandemic H1N1 human IAV strain from 2009, and their suckling piglets, exhibited high NTs against the most recent human H1 A vaccine strain. However, these titers are lost in piglets with waning maternally derived antibodies. Therefore, weaned piglets likely became more susceptible to H1 A IAV infection (see Fig. 2B). Indeed, the single human-to-pig transmission identified in this study was an H1A.3.3.2 strain. Neutralizing activity in porcine sera was also detected against recent human H3 vaccine strains. A wider reactivity was evident against the human vaccine strain A/Cambodia/e0826360/2020 compared to A/Darwin/9/2021. Recent sporadic transmissions of IAV from human to swine have been reported from Denmark and the US. However, the spread of the virus within swine populations appears to be impeded [47,48]. This phenomenon may be attributable to the presence of cross-reactive immunity within the swine population involving also other IAV proteins (e.g. the neuraminidase) as targets [49]. Furthermore, the presence in swine populations of antigenically distinct hemagglutinin (HA) proteins from an avian source could lead to new reassortants with the potential to cause a pandemic [50]. The highly pathogenic avian influenza A (HPAI) H5N1 virus, prevalent among wild birds and poultry, is a particular concern in this regard. Reports of natural HPAIV infections in domestic pigs already raised concern [24]. However, experimental studies have demonstrated a low degree of susceptibility to HPAIV H5N1 exposure, even at high doses of infection [51,52].

Molecular analyses of swIAV sequences established here confirm and extend previous data [6] that swIAVs circulating in Europe have already established human MxA resistance. This has been identified as a trait exhibited by all animal IAV that have resulted in a human pandemic [53]. MxA resistance was also detected in sequences of two recent zooanthroponotic human cases from swine holdings in Germany (Table 3). The zoonotic cases (MWP/21 and NRW/22) were not included in the study's systematic sampling. Instead, they were independently reported public health incidents that were analyzed retrospectively here. However, for one of the human cases, MWP/21, a matching porcine virus (swMWP/21) was isolated here from pigs originating from the holding where the human case occurred. Comparing the two sequences (Table S3) revealed that coding mutations were restricted to the M2 protein: In MWP/21, a triplet of mutations associated with adamantane resistance was found (L26I, V27A, S31N), while swMWP/21 revealed two mutations (L26I, V27A). The second human-derived sequences (NRW/22) showed M2 S31N. The NRW/22 case lacked verified swine contact, so no matching swine samples were collected. Adamantane resistance mutations are frequently detected independently of adamantane treatment and have become fixed in several M gene segment lineages of animal and human IAV [54,55].

## 5 Conclusions

In summary, the present study has demonstrated that pre-existing immunity, at least in part derived from neutralizing antibodies, may serve as a more substantial impediment to IAV transmission at the human-swine interface than previously assumed. Despite the wide interface that both species share and the presence of human MxA resistance mutations in swIAV, cross-species spillover infections of swIAV remain rare events. The further mitigation of risks associated with human exposure is contingent upon the enhancement of swIAV infection control measures in pigs. This, in turn, necessitates the implementation of advanced vaccines and vaccination strategies. Furthermore, the implementation of educational initiatives targeting swine farm staff and the dissemination of information regarding the potential benefits of seasonal IAV vaccination of farm personnel are recommended.

The following are the supplementary data related to this article.

Supplementary Fig. S1 Phylogenic tree of swIAV H1 HA gene of the clades 1A, 1B and 1C annotated by global H1-lineage nomenclature by Anderson et al. [13]. Supplementary Fig. S1

Supplementary Table S1 Summary of information of swine holdings and RT-qPCR results. Supplementary Table S1

Supplementary Table S2 Summary of information about human samples. Supplementary Table S2

Supplementary Table S3 Comparison of relevant mutations in the genome of MWP/21, swine-MWP/21, and NRW/22 generated by Flusurver (http://flusurver.bii.a-star.edu.sg). Supplementary Table S3

Supplementary Table S4 Accession number (EPI_ISL) of sequences analyzed in the frame of this study. Supplementary Table S4

Supplementary Table S5 Amino acids on positions in the nucleoprotein (NP) sequence associated with MxA resistance and BTN3A3 resistance of selected swIAV NP sequences. Supplementary Table S5

Supplementary material 1: Questinonaire for human participants. Image 1

Supplementary material 2: Questinonaire for swine farms. Image 2

Supplementary material 3: Materials & Methods, details. Image 3

## CRediT authorship contribution statement

**Christin Hennig:** Writing – original draft, Visualization, Methodology, Investigation, Formal analysis, Data curation. **Annika Graaf-Rau:** Writing – review & editing, Methodology, Investigation, Formal analysis, Data curation. **Kathrin Schmies:** Writing – review & editing, Methodology, Investigation, Formal analysis. **Roland Elling:** Writing – review & editing, Validation, Resources. **Philipp Henneke:** Writing – review & editing, Validation, Resources. **Ralf Dürrwald:** Writing – review & editing, Validation, Resources. **Elisabeth grosse Beilage:** Writing – review & editing, Validation, Resources. **Martin Schwemmle:** Writing – review & editing, Supervision, Project administration, Funding acquisition, Conceptualization. **Martin Beer:** Writing – review & editing, Supervision, Project administration, Funding acquisition, Conceptualization. **Timm Harder:** Writing – review & editing, Writing – original draft, Visualization, Validation, Supervision, Project administration, Methodology, Investigation, Funding acquisition, Data curation, Conceptualization.

## Ethical statement

Ethical approval was granted by Human Ethics Committees at the University of Greifswald, Germany, for sampling personnel at swine farms, and at the University of Freiburg, Germany, for the use of pediatric sera. Animals were investigated purely on basis of diagnostic approaches; therefore, no distinct approval was required from Animal ethics committees. Permission to sample blood from pigs in the frame of the PIGIE project has been granted by independent ethics committees in the German Federal States of Mecklenburg-Western-Pomerania (LALFF M-V 7221.3-2-004/22), Lower Saxony (LAVES NI 33.19-42502-04-22-00225) and North-Rhine-Westphalia (LANUV NW 81-02-04.40.2022.VG007).

## Declaration of competing interest

The authors declare that they have no known competing financial interests or personal relationships that could have influenced the work reported in this paper.

## Data Availability

Supplement materials are accessible under https://zenodo.org/records/14601669 [28]. Sequence data have been submitted to the GISAID EpiFlu database.
