# Supplementary figures and images for: High serological barriers may contribute to restricted Influenza-A-virus transmission between pigs and humans
Source: One Health. 2025 Oct 14;21:101214. doi: 10.1016/j.onehlt.2025.101214 (PMC12555762; doi:10.1016/j.onehlt.2025.101214)

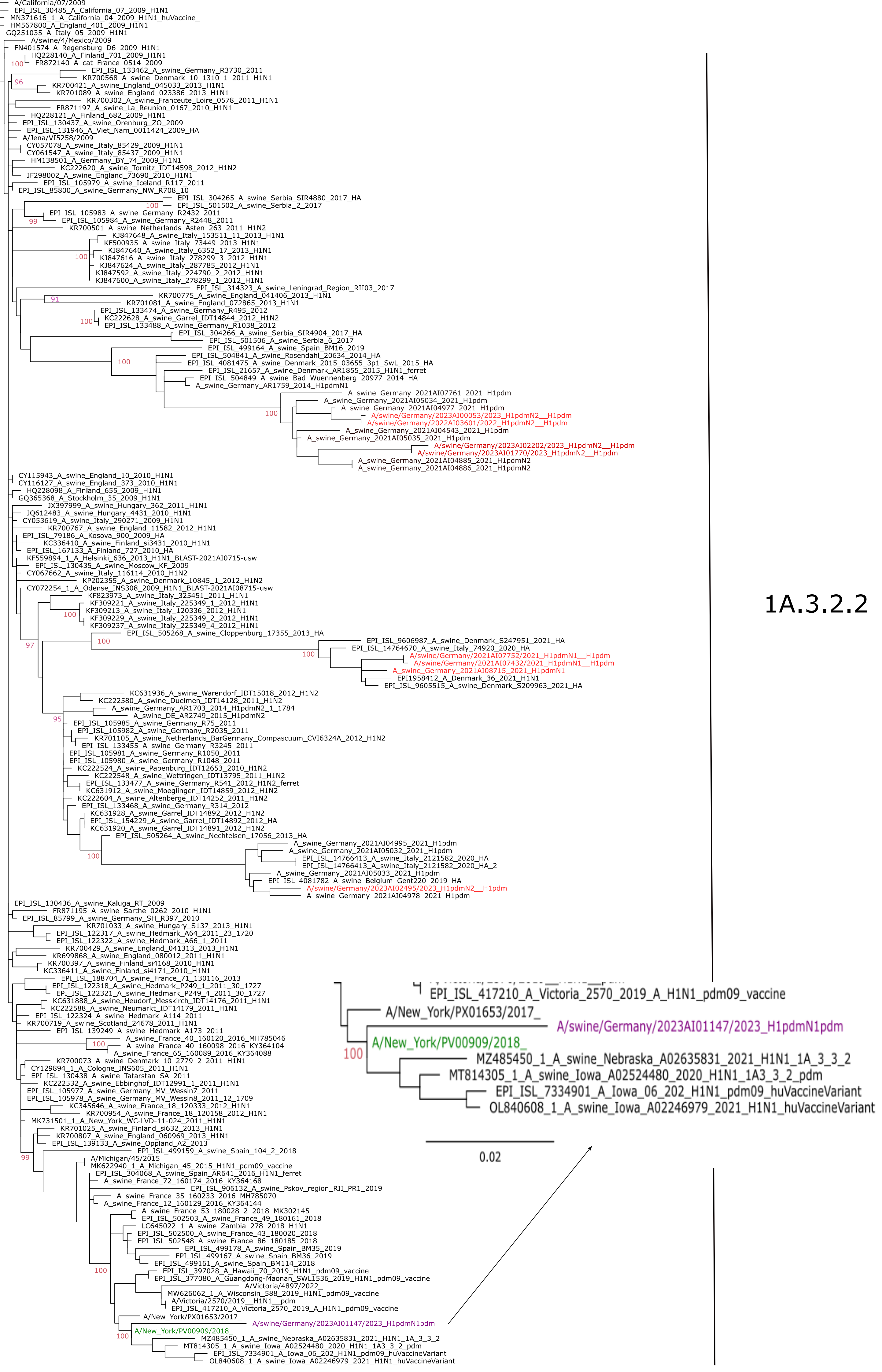

A/Hong\_Kong/117/1977

0.04

1B.1.1

1B.1.2.

1B.1.2.1

1B.1.2.3

1B.1.2.2

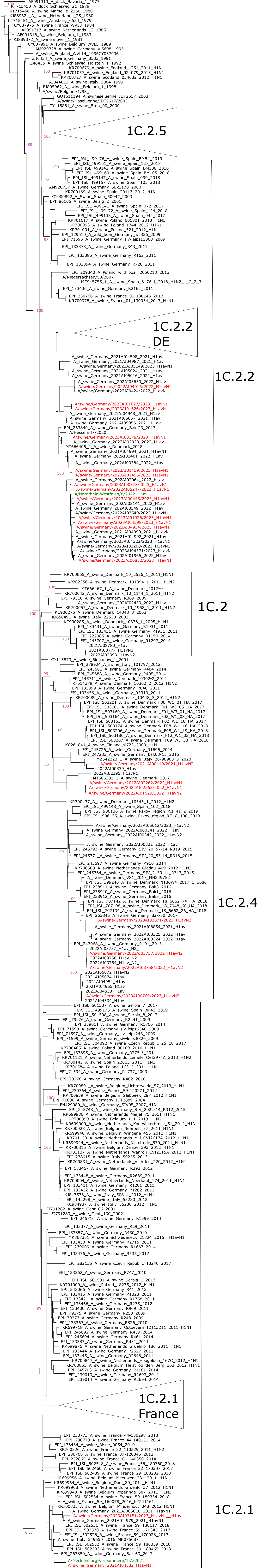

Supplement: Supplementary Fig. S1 — Phylogenic tree of swIAV H1 HA gene of the clades 1A, 1B and 1C annotated by global H1-lineage nomenclature by Anderson et al. [13]. [file mmc1.pdf]
