# Supplementary material for: High serological barriers may contribute to restricted Influenza-A-virus transmission between pigs and humans
Source: One Health. 2025 Oct 14;21:101214. doi: 10.1016/j.onehlt.2025.101214 (PMC12555762; doi:10.1016/j.onehlt.2025.101214)
Supplement: Supplementary file 7 — Supplementary material 1: Questinonaire for human participants. [file mmc7.pdf]

Supplemental Material

1. Technical Notes to Material and Methods

1.1 Virology

Origin of reference viruses

Swine influenza A viruses (swIAV) used as references here were obtained from collections at FLI or from submissions of this study. Human influenza A viruses (huIAV) representing seasonal vaccine strains, were provided by the National Influenza Centre at the Robert-Koch-Institute (RKI), Berlin, Germany.

Viral RNA extraction

RNA extraction was performed either manually by using the QIAamp Viral RNA Mini Kit (Qiagen, Hilden, Germany) or automatedly with the King Fisher Flex Purification System (Thermo Fisher Scientific) and the NucleoMag® Vet Kit (Macherey-Nagel GmbH & Co. KG, Dueren, Germany) according to the manufacturers' instructions.

Real time RT-PCR (RT-qPCR)

Swine samples were tested in a triplex-pathogen RT-qPCR assay established by Graaf-Rau et al. (2023) probing simultaneously for swIAV, porcine respirovirus-1 (PRV-1) and swine ortho-pneumovirus (SOV) [1]. Samples with cq-values ≤39.9 were considered as positive and further analyzed in a multiplex swIAV-subtyping RT-qPCR assay as previously described distinguishing five HA subtypes (H1av, H1hu, H1pdm, H3-84, H3-04) and three NA subtypes (N1av, N1pdm, N2) [1]. Human samples were analyzed exclusively for IAV by a generic M gene-specific RT-qPCR [2]. All RT-qPCR reactions were prepared with the AgPath-ID™ One-Step RT-qPCR kit (Thermo Fisher Scientific, United States) and run on a Biorad CFX96 Real-Time Cycler (Biorad, Germany).

Virus isolation in cell culture

Virus isolation was attempted for RT-qPCR-positive samples with a cq-value of ≤30. Madin-Darby-Canine kidney cells (MDCK-II) or swine testicle (ST) cells (Cell Bank at Friedrich-Loeffler-Institute, no. 0606) were employed as described previously [1].

Sequencing of swIAV genomes

Sanger sequencing was used to generate sequences of the HA IAV-gene from samples with cq-values ranging from 25-32 [1]. Field samples with cq-values ≤25 were selected for whole genome sequencing by Nanopore technology as described previously [3]. Sequences obtained were deposited in the EpiFlu database of GISAID. Accession numbers are presented in Table S2.

Phylogenetic analyses

HA segment-specific multiple alignments were generated using MAFFT (v7.450) [4] and manually curated and trimmed by AliView [5]. Phylogenetic estimations were carried out by maximum likelihood (ML) algorithms implemented in IQTree [6] utilizing ModelFinder included in IQTree to select the most appropriate model according to the Bayesian informative criterion [30]. Robustness of consensus trees was estimated using UFBoot [7] and trees were visualized with FigTree (V1.4.4) (http://tree.bio.ed.ac.uk/software/figtree/) and further manually edited with Inkscape (https://inkscape.org/). In addition, HA clades were determined using the BV-BRC tool [8] accessible via the BV-BRC website (www.bv-brc.org/app/SubspeciesClassification).

Genotyping

Genotyping was conducted following the approach of Graaf-Rau et. al (2023) [1] by aligning full length segmental swIAV sequences to reference sequences.

Molecular in silico analyses

The Flusurver online tool (http://flusurver.bii.a-star.edu.sg) was used to detect and analyze mutations in the IAV genome. Neutralization-relevant epitopes in deduced HA1 protein sequences of swIAV and human IAV were compared according to Sun et al. (2020) [9]. Alignments were generated with MAFFT using the Geneious software version 2021.0.1 and further processed with WebLogo [10] and Biorender.com (https://www.biorender.com/). Mutations in the NP protein that interfere with Mx and BTN3A3 factors were identified according to Henritzi et al. (2020) [11] and Pinto et al. (2023) [12].

1.2 <u>Serology</u>

Enzyme linked immunosorbent assay (ELISA)

Serum samples were heat-inactivated (56°C for 30 min) before first use. For the human sera, the ab108745 - Anti-Influenza virus A IgG Human ELISA Kit (Abcam®, Cambridge, United Kingdom) was used to detect IgG antibodies against IAV following the manufacturer's instructions. The swine sera were tested for IAV NP-specific antibodies by the ELISA kit ID Screen Influenza A Antibody Competition Multi-species (IDvet®, Grabels, France) according to the manufacture's protocol.

Virus neutralization assay (VNT)

Heat-inactivated human and swine serum samples were treated with neuraminidase as previously described [11]. MDCK-II cells were seeded into 96-well cell culture plates and incubated in cell growth medium (DMEM, 5% fetal calf serum, FCS) at 37°C overnight to allow forming an 80-90% confluent monolayer. Serum samples were serially diluted twofold, starting at 1:20, in DMEM medium supplemented with 6-(1-tosylamido-2-phenyl)-ethyl-chloromethyl-ketone (TPCK)-treated trypsin (infection medium) at a final concentration of 1 µg/mL. Viruses used in this study, were diluted in infection medium to a concentration of 2,000 $TCID_{50}$/ml ($10^{3,3}$ $TCID_{50}$/ml). At a volume of 50 µl each, diluted serum and virus were mixed and incubated for 1 h at 37°C. The serum/virus mixture was then transferred to the MDCK-II cell plates, from which growth medium had been removed and monolayers washed once with PBS. The plates were incubated at 37°C for 72 h, after which cytopathic effects (CPE) were recorded. Virus titrations were performed in parallel to ensure the virus amount had been set to the correct $TCID_{50}$ ($10^{3,3}$ $TCID_{50}$/ml; i.e. 100 $TCID_{50}$ per well).

Immuno-peroxidase monolayer assay (IPMA)

IPMA was performed to visualize IAV antigen in cell cultures using a peroxidase-labelled (POD) antibody for improved assessment of the VNT in addition to CPE readout. Medium was removed from MDCK-II cell cultures and monolayers carefully washed with PBS diluted 1:2 with bidistilled water. Wash fluid was removed, plates air-dried and then heat-fixed at 80°C for 4 h. Hybridoma culture supernatant containing monoclonal antibody specific for the nucleocapsid protein of IAV (mAb 890, H16-L20-5R5, FLI Biobank) was diluted 1:50 with undiluted PBS to which 0.005% Tween 20 has been added (PBST) and incubated on heat-fixed cells at 37°C for 1 h. Thereafter cells washed 3 times with PBST. The secondary antibody, a POD-antispecies goat anti-mouse IgG (H/L) HRP conjugate (Bio-Rad Laboratories GmbH, Feldkirchen, Germany) was diluted 1:500 with PBST and transferred onto the fixed cells, with an incubation time of 1 h at 37°C. Fixed cells were washed again 3 times with PBST, afterwards incubated with bidistilled water for 10-15 minutes and then discharged. The fixated cells were finally incubated with a precipitating, chromogenic substrate (3-Amino-9-Ethylcarbazol, AEC) in sodium acetate buffer to which H_2O_2 had been added. After an incubation period of 15-30 minutes, the antigen-antibody reaction was assessed as a brownish intracellular precipitate by light microscopy.

2. References

1. Graaf-Rau, A., C. Hennig, K. Lillie-Jaschniski, et al., *Emergence of swine influenza A virus, porcine respirovirus 1 and swine orthopneumovirus in porcine respiratory disease in Germany.* Emerg Microbes Infect, 2023. **12**(2): p. 2239938. DOI: 10.1080/22221751.2023.2239938.

2. Hassan, K.E., A.K. Ahrens, A. Ali, et al., *Improved Subtyping of Avian Influenza Viruses Using an RT-qPCR-Based Low Density Array: 'Riems Influenza a Typing Array', Version 2 (RITA-2).* Viruses, 2022. **14**(2). DOI: 10.3390/v14020415.

3. King, J., T. Harder, M. Beer, et al., *Rapid multiplex MinION nanopore sequencing workflow for Influenza A viruses.* BMC Infect Dis, 2020. **20**(1): p. 648. DOI: 10.1186/s12879-020-05367-y.

4. Katoh, K., J. Rozewicki, and K.D. Yamada, *MAFFT online service: multiple sequence alignment, interactive sequence choice and visualization.* Brief Bioinform, 2019. **20**(4): p. 1160-1166. DOI: 10.1093/bib/bbx108.

5. Larsson, A., *AliView: a fast and lightweight alignment viewer and editor for large datasets.* Bioinformatics, 2014. **30**(22): p. 3276-8. DOI: 10.1093/bioinformatics/btu531.

6. Nguyen, L.T., H.A. Schmidt, A. von Haeseler, et al., *IQ-TREE: a fast and effective stochastic algorithm for estimating maximum-likelihood phylogenies.* Mol Biol Evol, 2015. **32**(1): p. 268-74. DOI: 10.1093/molbev/msu300.

7. Minh, B.Q., M.A. Nguyen, and A. von Haeseler, *Ultrafast approximation for phylogenetic bootstrap.* Mol Biol Evol, 2013. **30**(5): p. 1188-95. DOI: 10.1093/molbev/mst024.

8. Olson, R.D., R. Assaf, T. Brettin, et al., *Introducing the Bacterial and Viral Bioinformatics Resource Center (BV-BRC): a resource combining PATRIC, IRD and ViPR.* Nucleic Acids Res, 2023. **51**(D1): p. D678-D689. DOI: 10.1093/nar/gkac1003.

9. Sun, H., Y. Xiao, J. Liu, et al., *Prevalent Eurasian avian-like H1N1 swine influenza virus with 2009 pandemic viral genes facilitating human infection.* Proc Natl Acad Sci U S A, 2020. **117**(29): p. 17204-17210. DOI: 10.1073/pnas.1921186117.

10. Crooks, G.E., G. Hon, J.M. Chandonia, et al., *WebLogo: a sequence logo generator.* Genome Res, 2004. **14**(6): p. 1188-90. DOI: 10.1101/gr.849004.

11. Henritzi, D., P.P. Petric, N.S. Lewis, et al., *Surveillance of European Domestic Pig Populations Identifies an Emerging Reservoir of Potentially Zoonotic Swine Influenza A Viruses.* Cell Host Microbe, 2020. **28**(4): p. 614-627 e6. DOI: 10.1016/j.chom.2020.07.006.

12. Pinto, R.M., S. Bakshi, S. Lytras, et al., *BTN3A3 evasion promotes the zoonotic potential of influenza A viruses.* Nature, 2023. **619**(7969): p. 338-347. DOI: 10.1038/s41586-023-06261-8.
