# Supplementary material for: High serological barriers may contribute to restricted Influenza-A-virus transmission between pigs and humans
Source: One Health. 2025 Oct 14;21:101214. doi: 10.1016/j.onehlt.2025.101214 (PMC12555762; doi:10.1016/j.onehlt.2025.101214)
Supplement: Supplementary file 8 — Supplementary material 2: Questinonaire for swine farms. [file mmc8.pdf]

Isolation of influenza viruses in pigs and humans

Personal data sheet

Nasal swab collection (date): ____ . ____ . _______ **Labelling:** ________

Angaben zur Person:

Surname, Name:____

Birth date:____

Street adress: ____

City code; City: ____

E-Mail or Tel.-no: ____

Do you currently have the following symptoms?

		Onset of symptoms (date):
**Fever**	☐	
**Cough**	☐	
**Nasal discharge**	☐	
**Headache and/or aching limbs**	☐	
**Other:**		

Have you ever had a flu vaccination?

No ☐ Yes ☐

If yes, how often did you receive vaccination?

Regularly (annually) ☐

Irregularly ☐

What is your connection to pig farming?

Employee at swine farm	Veterinarian	Family member of Employee	Family member of veterinarian
☐	☐	☐	☐

Information on the workplace

Company/Veterinary practice: ____

Street adress:____

City code; City: ____

Date: __________________________ Signature: ___________________________________
