# Supplementary material for: High serological barriers may contribute to restricted Influenza-A-virus transmission between pigs and humans
Source: One Health. 2025 Oct 14;21:101214. doi: 10.1016/j.onehlt.2025.101214 (PMC12555762; doi:10.1016/j.onehlt.2025.101214)
Supplement: Supplementary file 9 — Supplementary material 3: Materials & Methods, details. [file mmc9.pdf]

Isolation of influenza viruses in pigs and humans

Swine farm questionaire

Nasal swab collection (date): _______________

Sampled age groups:

Sample-No.	Barn-No.	Suckling piglets	Piglets	Other: _____________
1		◻	◻	◻
2		◻	◻	◻
3		◻	◻	◻
4		◻	◻	◻
5		◻	◻	◻
6		◻	◻	◻
7		◻	◻	◻
8		◻	◻	◻
9		◻	◻	◻
10		◻	◻	◻
11		◻	◻	◻
12		◻	◻	◻
13		◻	◻	◻
14		◻	◻	◻
15		◻	◻	◻
16		◻	◻	◻
17		◻	◻	◻
18		◻	◻	◻
19		◻	◻	◻
20		◻	◻	◻

Current health status:

	Fever	Coughing	Anorexia	Infertility	Abortions	Weak piglets	Other
Sows	◻	◻	◻	◻	◻	◻	_________
Rearing	◻	◻	◻	◻	◻	◻	_________
Fattening pigs	◻	◻	◻	◻	◻	◻	_________
Onset, Duration of symptoms							

Influenza history:

Has there already been a proven influenza outbreak in the herd?

No □ Yes □ If yes, which subtype was detected? _________

Influenza vaccination:

	Vaccinated	Not vaccinated	Last vaccination on	Vaccine:
Gilts	□	□		
Sows	□	□		
Rearing/Fattening pigs	□	□		

Herd vaccination □ Reproductive-related vaccination □

Information about herd

**Company name:**	
**Specialisation of fram**	
**Number of pigs**	Piglets: ______ Sows: ______ Fattening pigs: ______
**Street adress:**	
**City code, City:**	
**Telefone number:**	
**E-Mail adress:**	

Veterianrian:

**Practice:**	
**Street adress:**	
**City code, City:**	
**Telefone number:**	
**E-Mail adress:**	

Date: ___________________________

Signature: ____________________________

□ **Company owner/Deputy manager**
□ **Veterinarian**
